# Whole heart 3 D MR coronary angiography with and without extracellular contrast agent

**DOI:** 10.1186/1532-429X-11-S1-P125

**Published:** 2009-01-28

**Authors:** Dorota Piotrowska-Kownacka, Lukasz Kownacki, Grzegorz Opolski, Leszek Krolicki, Olgierd Rowinski

**Affiliations:** Medical University of Warsaw, Warsaw, Poland

**Keywords:** Contrast Injection, Improve Image Quality, Microvascular Obstruction, Acute Myocarditis, Extracellular Contrast Agent

## Introduction

It is well known that extravascular contrast administration before whole heart 3 D MR angiography improves image quality and distal coronary segments visibility. In opposite to extravascular, extracellular contrast agents are commonly used in cardiac MR. The influence on coronary angiography quality remains unknown.

## Purpose

The aim of the study was quality assessment of coronary MR angiography performed after extracellular contrast administration (Gd-DTPA) in stable, consecutive patients referred to CMR lab for contrast enhanced cardiac examination in comparison to unenhanced coronary MR angiography in healthy volunteers.

## Methods

69 patients and 16 volunteers were examined in 1.5 T scanner with 3D navigator gated, inversion recovery, segmented gradient echo sequence. For data acquisition 32 Channel Cardiac Coil was used. In all patients MR angiography was the last sequence applied. In all patients extracellular contrast agent (Gd-DTPA, 0.1 mmol/kg b.w.) was injected. The time between contrast injection and whole heart MR angiography was monitored. The images were analyzed on MMWP Workstation. Quality of MR angiography was evaluated using 4 point scale: 1-poor, 2-sufficient, 3-good, 4-very good. General image quality and visibility of coronary artery disease: left main, proximal and distal parts of LAD, Cx, RCA were evaluated separately. Differences between unenhanced and enhanced data were analyzed using Mann-Whitney test. P value of below 0,05 was considered statistically significant.

## Results

Mean time between contrast injection and sequence start was 12,8 ± 7,7 min. Mean acquisition time was 14 ± 5,9. The median quality score was 3. General MR angiography quality was good or very good in 76,6% of patient and 67,7% of volunteers. There was significant improvement of general quality as well as coronaries visibility in all evaluated arteries and parts on enhanced images. No relation between contrast injection time as well as acquisition time and image quality were found. Enhanced images have given additional information regarding viability in patients with acute myocarditis and viability/no-reflow regions in patients early after myocardial infarction.

## Conclusion

**Figure 1:**
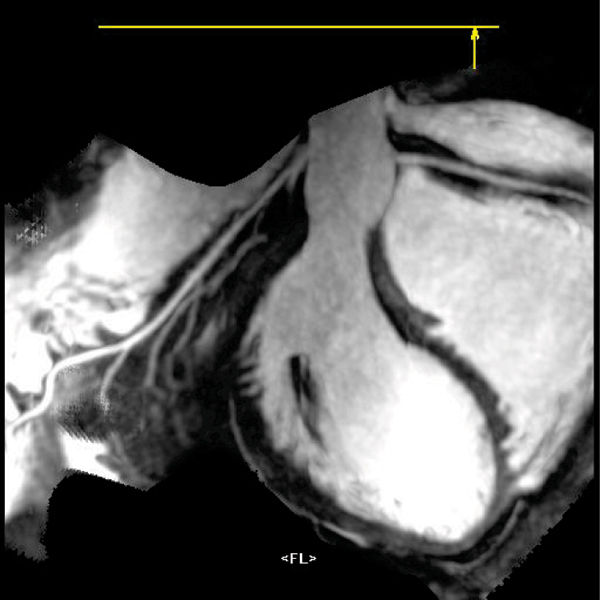
**Enhanced MR coronary angiography – normal coronaries**.

**Figure 2:**
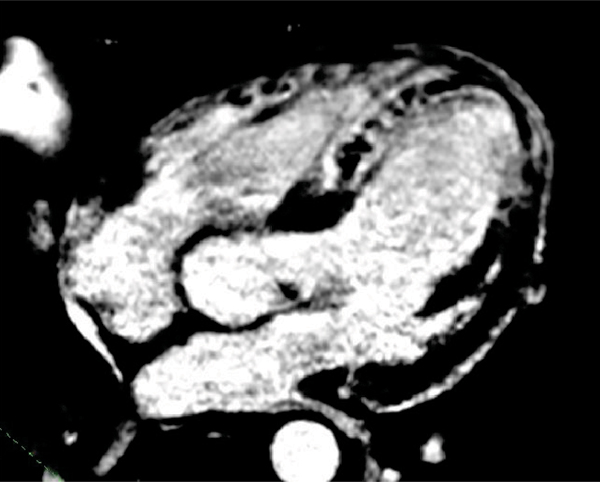
**DE and MVO in STEMI**.

**Figure 3:**
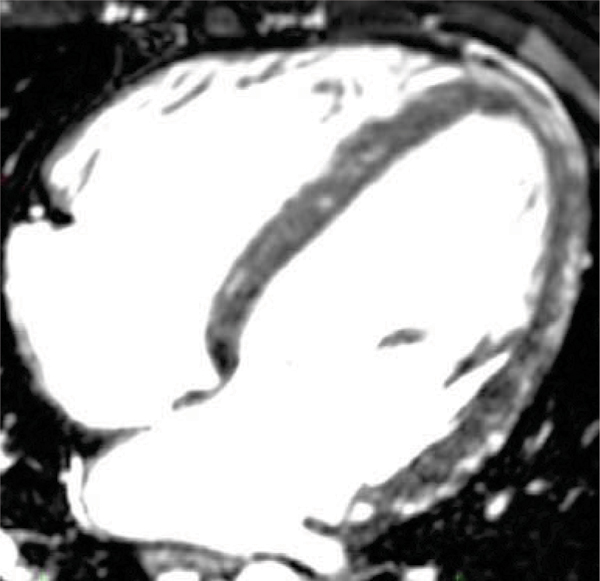
**DE in myocarditis**.

Extracellular contrast agents, which are commonly used in cardiac MR, significantly improves image quality of the whole heart, navigator gated 3D MR coronary angiography. Preliminary data suggests that this sequence could be used as "one stop shop" alternative in patients in acute condition uncapable of breath-hold. MR angiography, morphology as well as viability and microvascular obstruction regions could be imagined. Further studies are needed. Please refer to Figures 1, 2, and 3.

